# P-1999. Predictors of Mortality in Myelodysplastic Syndrome with COVID-19: A 2020-2021 National Study

**DOI:** 10.1093/ofid/ofae631.2156

**Published:** 2025-01-29

**Authors:** Madhumithaa Jagannathan, Barath Prashanth Sivasubramanian, Sonia Babu, Shanthi Reddy Sripathi, Avinash Javvaji, Priyanshu Jain, Shashvat Joshi, Dinesh Kumar Shanmugam, Bharath Duraisamy Swami Kannan, Rutul Dalal, Raghavendra Tirupathi

**Affiliations:** M.I.M.E.R Medical College, Talegaon Dabhade, Pune, Maharashtra, India, Chambersburg, Pennsylvania; University of Texas Health San Antonio, San Antonio, Texas; Ramaiah Medical College, Bangalore, Karnataka, India, Chambersburg, Pennsylvania; Osmania Medical College, Hyderabad, Telangana, India, Hyderabad, Telangana, India; Chalmeda Anandrao Institution of Medical Sciences, Karimnagar, Telangana, India, Chambersburg, Pennsylvania; Kasturba Medical College, Manipal, Udupi, Karnataka, India, Chambersburg, Pennsylvania; Shanghai Medical College, Fudan University, Shanghai, China, Chambersburg, Pennsylvania; PSG IMSR, Avinashi Road, Peelamedu, Coimbatore, Tamil Nadu, India, Chambersburg, Pennsylvania; Government Sivagangai Medical College, Sivaganga, Tamil Nadu, India, Chambersburg, Pennsylvania; Medical Director, Penn State Health (Eastern Region), Penn State Health St. Joseph Medical Center, Pennsylvania, USA, Lancaster, Pennsylvania; Keystone Health, Chambersburg, Pennsylvania, USA, Chambersburg, Pennsylvania

## Abstract

**Background:**

We aimed to evaluate mortality predictors in Myelodysplastic Syndrome who were admitted for COVID-19 (MDSCov) using the Healthcare Cost and Utilization Project.

Sociodemographics in MDSCoV Patients

**Figure:**
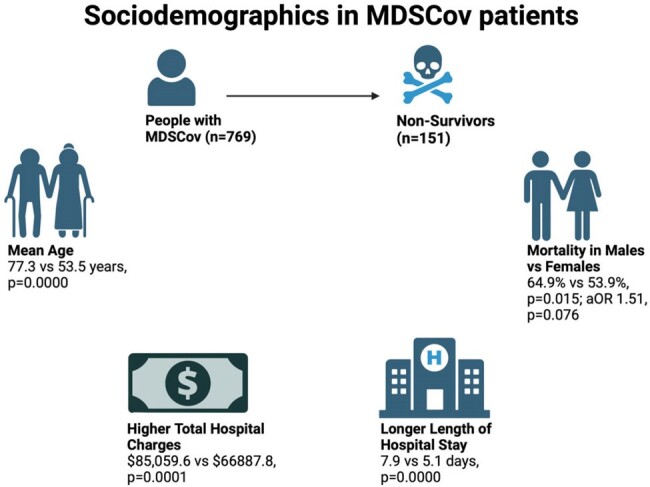

**Methods:**

A cross-sectional study using the NIS on adults (≥ 18 years) hospitalized in the United States for MDSCov (n=769). We identified 151 nonsurvivors among MDSCov. Analysis was done using t-tests and chi-square tests, and multivariate regression models were constructed with p≤ 0.05 significance.

Factors Associated with Higher Mortality in MDSCoV Patients

**Figure:**
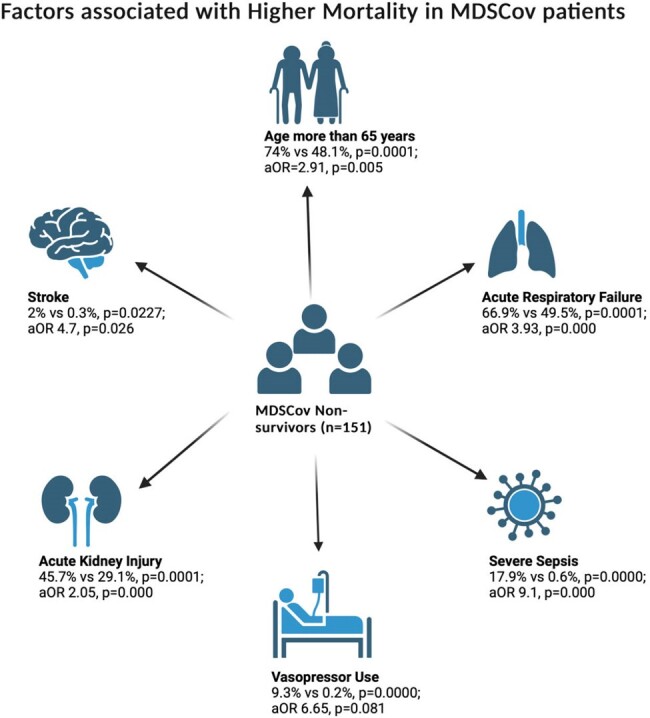

**Results:**

The average age of MDSCov was higher than those without COVID-19 (77.3 vs 53.5 years, p=0.0000). MDSCov had a longer average length of stay (7.9 vs 5.1 days, p=0.0000) and higher total hospital charges ($85,059.6 vs $66887.8, p=0.0001) compared to MDS patients without COVID-19. We found that HSCT (allogenic and autogenic) was not associated with increased MDSCov hospitalization (2.2% vs 2.5%, p=0.8286). Males experienced a higher mortality rate (64.9% vs 53.9%, p=0.015; aOR 1.51, p=0.076) as they have higher expression of ACE2 receptors than females, correlating to disease severity. Race did not contribute to mortality (White 72.8% vs 77.3%, p=0.5267). The Non Survivors of MDSCov showed a high association with stroke (2% vs 0.3%, p=0.0227; aOR 4.7, p=0.026), severe sepsis (17.9% vs 0.6%, 0.0000; 9.1, 0.000), acute respiratory failure (66.9% vs 49.5%, 0.0001; 3.93, 0.000), and acute kidney injury (45.7% vs 29.1%, 0.0001; 1.27, 0.326). Due to COVID-19, T-cell exhaustion and secondary infections that contribute to sepsis can occur. Increased microthrombus formation and endothelial dysfunction can lead to thromboembolic episodes. Increased proinflammatory cytokines (IL-1β, IL-6, IL-12, IFN-γ, IP-10, and MCP-1) caused by the virus contribute to pulmonary inflammation and lung injury. The use of vasopressors to maintain MAP (9.3% vs 0.2%, p=0.0000; aOR 6.65, p=0.081) was markedly higher but not significant among nonsurvivors.

Sociodemographic Profile of Patients with MDS

**Figure:**
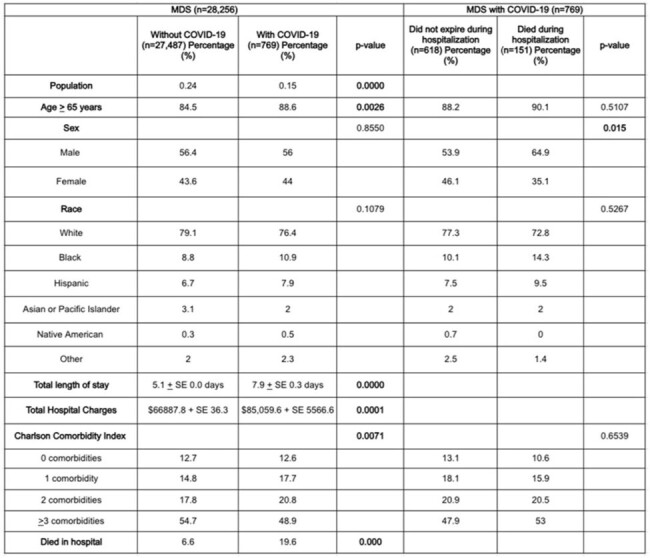

**Conclusion:**

Our study identified several predictors of mortality in MDSCov. However, due to the need for more information on disease severity and laboratory evidence, future studies are needed to develop timely treatment plans. Appropriate chemotherapeutic regimens for MDS and antivirals/biologics for COVID-19 are needed.

Predictors of In-hospital Mortality in Patients with MDS and COVID-19

**Figure:**
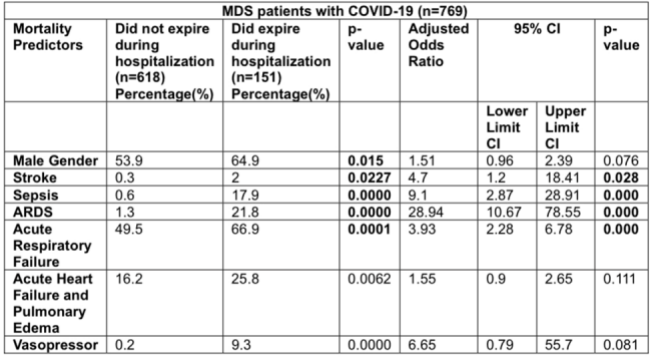

**Disclosures:**

All Authors: No reported disclosures

